# Supplementary material for: The quality of life and well-being of nursing home residents and their relatives during the COVID-19 pandemic: A quantitative approach
Source: PLOS Ment Health. 2025 Jan 29;2(1):e0000233. doi: 10.1371/journal.pmen.0000233 (PMC12798449; doi:10.1371/journal.pmen.0000233)
Supplement: Attachment — Submitted filename: PMEN-D-24-00288 Response to Reviewers.docx [file pmen.0000233.s001.docx]

PMEN-D-24-00288

The quality of life and well-being of nursing home residents and their relatives during the COVID-19 pandemic: a quantitative approach

Rebutal Letter

*Please include the following items when submitting your revised manuscript:*

 *A rebuttal letter that responds to each point raised by the editor and reviewer(s). You should upload this letter as a separate file labeled 'Response to Reviewers'.*

 *A marked-up copy of your manuscript that highlights changes made to the original version. You should upload this as a separate file labeled 'Revised Manuscript with Track Changes'.*

 *An unmarked version of your revised paper without tracked changes. You should upload this as a separate file labeled 'Manuscript'.*

1. Review Comments to the Author

Please use the space provided to explain your answers to the questions above. You may also include additional comments for the author, including concerns about dual publication, research ethics, or publication ethics. (Please upload your review as an attachment if it exceeds 20,000 characters)

Reviewer #1:

One of the aims discussed in the introduction is assessing coping mechanisms to cope with COVID-19 measures, but the mechanisms are not much discussed in the results or discussion.

*Answer: Section added p.16 to discuss coping mechanism more thoroughly*

The introduction covers that both residents and their relatives are impacted by protection measures. Why would a comparison between the two groups be necessarily needed?

Both samples of residents and their relatives, especially the relatives, are heterogeneous groups that would benefit from a more nuanced interpretation of results. For example, if relatives were found to have a higher hyperarousal score than residents, what do these results mean and how can this finding benefit the literature?

*Answer: Since both samples lived in the same socio-cultural context, difference between sample can more easily be attributed to specific about the population. We emphasized this in the introduction (p.5-6)*

The current analyses may be too general for the results to be truly meaningful. If possible, it would be interesting to compare each group before and after the COVID-19 measures were implemented or to include how long each resident has been in the nursing home to see if perhaps the length of stay may influence their coping or quality of life.

*Answer: Quantitative data about nursing home resident’s wellbeing were not routinely gathered in Switzerland before the COVID-19 pandemic. Thus, we unfortunately do not have pre-covid data to compare our results with. However, the opposite analysis can also be done. Thus, referencing data about residents’ wellbeing during an event such as COVID-19 could be used as a base of comparison with similar data during less stressful time. This is partly why our data are also referenced on a data repository (osf.io). In addition, median length of stay of nursing home residents in Switzerland is around 3 years, with little variation. Thus, adding this factor would not add significant explaining factors in our sample. We expended the discussion section to talk about that.*

The finding that residents’ PSS scores negatively impacted their QoL scores is interesting but also somehow logically expected. The authors say that these were congruent with the idea that lockdown measures mainly impacted residents’ perceived stress. How were residents' perceptions of lockdown measures incorporated into the questionnaire? In turn, were other aspects that heavily influence QoL (e.g., physical health, culture, etc.) measured? If not, what are some potential directions for future studies?

*Answer: This article presents the quantitative results of a mixed-methods project. Participants that responded to the questionnaire also participated in semi-directed interviews (Cohen et al. 2024). In this paper, residents clearly describe protection measure having a direct impact on their wellbeing. We elaborated more on this in the discussion section.*

An additional limitation is the fact that the sample came from one nursing home. More description of the nursing home (how big it is, whether it is located in the urban or countryside, and what specific measures were implemented and remained after 1 year) would be a good addition.

*Answer: Description of the 13 nursing home from in which participants were located can be found in the method section (under “population”). We never stipulated that they were all coming from the same nursing home.*

The paper would benefit from some suggestions on how to design new protection measures that take QoL into account. Some added literature into the discussion on what interventions have been proposed or implemented to help alleviate perceived stress from a pandemic and its following lockdown measures would help the paper be more fruitful.

*Answer: We've added a paragraph detailing the recommendations arising from the qualitative part of the recently published study. we've also added a paragraph detailing the recommendations arising from a project commissioned by the Swiss Federal Office Of Public Health which gathered testimonials from residents and their families. a panel of experts was able to establish a series of postulates and recommendations to better balance life protection measures and maintaining quality of life for a new pandemic (p.19).*

Reviewer #2: PMEN-D-24-00288

The quality of life and well-being of nursing home residents and their relatives during the COVID-19 pandemic: a quantitative approach

- It is important that authors include some discussion of the existing literature on the relationship between isolation and wellbeing or quality of life. To say that “…there was a chance they (measures against COVID-19) could have a detrimental impact on residents’ quality of life” is to ignore any reference to existing literature.

*Answer: The impact of social isolation on mental health is discussed more thoroughly in the introduction, with added recent literature (p.4).*

- P. 5: “The present study used a questionnaire measuring three domains of symptomatology that can develop out of post-traumatic stress disorder: Intrusion, Avoidance and Hyperarousal.” It is my understanding that these domains do not develop out of PTSD, but are central to the diagnosis itself. So, the statement needs to be clarified. The authors should explain the rationale for choosing measures that only focus on Intrusion, Avoidance and Hyperarousal symptom clusters. The DSM-5 diagnostic criteria for PTSD includes many more components that these. It is important for the authors to provide a rationale for their decision.

*Answer: Posttraumatic Stress Disorder (PTSD) can manifest in various symptoms that do not necessarily meet the full diagnostic criteria. Indeed, individuals may experience distressing memories or flashbacks related to the trauma, albeit less frequent or intense than those seen in full PTSD cases (French et al., 2024). Avoiding reminders of the trauma, such as places or conversations, can occur, but may not be pervasive enough to meet full criteria (Mordeno & Luzano, 2023) and symptoms such as irritability or heightened startle response can be observed, though they may not be as severe (Schmidt, 2015). While these subthreshold symptoms can significantly impact an individual's quality of life, they may not be recognized as PTSD, leading to potential under-treatment.*

*A paragraph was added to provide a rational for this decision (p. 5-6).*

- With regard to instruments used, it is important to provide the psychometric properties of any measures used. All measures used to gather quantitative data appear to be standardized. The authors should, therefore, provide relevant psychometric properties/information (reliability and validity) related to each of the measures.

*Answer: Cronbach’s alpha were integrated in the text, in the “scale used and data gathering” section*

- p. 10: I find this statement a bit confusing: “The mean resident and relative scores for the questionnaires and scales that both groups responded to were compared, and the t-test was used to quantify potential differences between the samples.” Perhaps, it may be written thus: The mean scores on questionnaires completed by both groups (residents and relatives) were compared, and a t-test was used to measure potential differences between the groups.

*Answer: Thank you for the rephrasing, it is indeed clearer (changes made in the main text).*

- p. 10: I do not see a need to include any description of the General Health Questionnaire (GHQ), since it was not used for any reported data in this study/manuscript.

*Answer: all aspects of the main text related to GHQ was removed from manuscript.*

- The data analysis section needs some more clarifying description. Notably, it is important for the authors to explain what “requirements for parametric statistical tests” the data did not meet? Additionally, it would be helpful for the reader to know some of the key assumptions of parametric statistical tests as these may provide insight into the authors’ decision-making and rationale. In other words, which of the three key assumptions (normal distribution, homogeneity of variance, and independence of the variable) did the sample data not meet?

*Answer: precisions were added (non-normal distribution).*

- The discussion section needs to be contextualized within existing literature. As it stands, lacks details of how the results (significant or not) may be interpreted/understood within the context of current or extant literature.
- *Answer: A paragraph have been added on p19*

Reviewer #3: ABSTRACT

In the Methods section of the abstract, specify the sample and the type of questionnaire used in this study. The results should report the key findings of the study based on the study objectives.

- *Answer: Abstract was changed to include the aforementioned information.*

INTRODUCTION

The introduction is well-written. However, I have few comments for your consideration.

‘Interestingly, few studies have jointly investigated the psychological well-being of residents and their relatives in the face of the measures implemented in nursing homes to prevent COVID-19 infection. ’Can you provide references for some of the studies?

*Answer: The text was corrected on p.4 to underline that this is a result from a review on the subject*

METHODS

‘The age of our participants ranged from 65 to 90+ years, with most participants being 80- 89 years old’

How did you establish the mental capacity of the population to respond to the questionnaire?

*Answer: participants also participated in the qualitative part of this subject, and thus an experimenter could directly assess the inclusion criteria. This has been clarified in the text*

“Our participants responded to a series of self-administered questionnaires investigating their own responses. perceived stress, quality of life, post-trauma growth, and coping responses to stressful events such as a lockdown or a pandemic”

Explain if the participants required assistance in responding to the questionnaires.

*Answer: it has been added that the experimenter was present when participants responded to the questionnaire and could ask the experimenter for clarification if needed.*

‘The only (minimal) difference between the groups was that resident’s quality of life was evaluated using WHOQOL-OLD, and relatives’ QoL was evaluated using the WHOQOL- BREF scale.’

Justify the use of different versions of the scale in this study.
*Answer: A justification have been added on p7*

DISCUSSION

The discussion can be improved by comparing the findings of this study with those of previous literature.

*Answer: A paragraph have been added on p19*
